# Low energy availability in recreational high-intensity functional training athletes: associations with health and performance outcomes

**DOI:** 10.3389/fnut.2026.1953641

**Published:** 2026-10-23

**Authors:** Gabriella Vlattas, Xuewen Wang, Andrew Ortaglia, Siqi Hong, Toni Torres-McGehee

**Affiliations:** 1 Athletic Training Research Lab, Department of Exercise Science, University of South Carolina, Columbia, SC, United States; 2 Department of Epidemiology and Biostatistics, University of South Carolina, Columbia, SC, United States

**Keywords:** bone mineral density, energy availability, exercise recovery, high-intensity functional training, low energy availability, relative energy deficiency in sport (RED-S)

## Abstract

**Introduction:**

Low energy availability (LEA) is the primary etiological factor underlying Relative Energy Deficiency in Sport (RED-S), yet limited research has examined LEA risk and associated health and performance outcomes in recreational high-intensity functional training (HIFT) athletes. The purpose of this study was to evaluate energy availability (EA) and physiological outcomes aligned with the RED-S Health and Performance Models in a recreational HIFT cohort.

**Methods:**

Thirty participants (females: *n* = 18, males: *n* = 12; 31.2 ± 8.2 years) completed a two-week prospective observational study with repeated measures of energy intake, exercise energy expenditure, and associated health and performance-related measures. Energy intake was assessed via dietary logs, exercise energy expenditure was estimated using a wearable armband, and EA was calculated relative to dual-energy X-ray absorptiometry–derived fat-free mass. Participants were classified as LEA if EA was <30 kcal·kg^−1^ FFM·day^−1^. Performance-related outcomes included rating of perceived exertion (RPE), perceived recovery status (PRS), and total quality recovery (TQR). Health-related outcomes included sleep duration, Pittsburgh Sleep Quality Index (PSQI), and bone mineral density (BMD) (reported by *Z*-Score).

**Results:**

Mean EA was 24.9 ± 9.7 kcal·kg^−1^ FFM·day^−1^, with 80% (*n* = 24) of participants demonstrating EA below the LEA classification threshold. The high prevalence of LEA should be interpreted cautiously given the potential for underreporting dietary intake. LEA status was not associated with differences in recovery, sleep quality, or BMD (*p* > 0.05), and EA was not significantly correlated with performance or health outcomes. In an exploratory analysis, sleep duration was moderately positively associated with TQR (*ρ* = 0.46, *p* = 0.01); however, this finding should be interpreted cautiously given the number of comparisons performed.

**Discussion:**

Despite the high prevalence of LEA, associated health and performance impairments were not detected, suggesting that measurable consequences of LEA may not be evident over a short observation period in recreational HIFT athletes. These findings support ongoing monitoring of EA, fueling practices, and recovery behaviors to reduce the potential long-term consequences associated with RED-S.

## Introduction

High-intensity functional training (HIFT) represents a rapidly growing training modality characterized by high-intensity, functional, and constantly varied exercise designed to improve multiple domains of fitness (1). HIFT typically incorporates resistance training, metabolic conditioning, and functional movement patterns performed at high intensity, resulting in substantial exercise energy expenditure (1, 2). While these training characteristics contribute to improvements in fitness and performance, they may also increase the risk of inadequate energy intake relative to physiological demands. Recreational HIFT athletes may be particularly vulnerable because they often balance demanding training schedules with occupational, academic, and family responsibilities while having limited access to professional nutrition support (2, 3). Additionally, the emphasis on lean physique and performance commonly observed within HIFT culture may encourage intentional or unintentional dietary restriction (2). Together, these factors may increase the risk of low energy availability (LEA) in this population.

Energy availability (EA) is defined as the amount of dietary energy remaining for normal physiological function after accounting for exercise energy expenditure, relative to fat-free mass (FFM) (4). When EA is sufficient, physiological systems operate optimally to support cellular maintenance, hormonal regulation, tissue repair, and adaptation to training stimuli. When energy intake is insufficient to support both exercise demands and essential physiological processes, a state of LEA occurs (5–7). Even short-term exposure to LEA can disrupt metabolic and endocrine function, while prolonged or repeated exposure increases the risk of chronic physiological dysfunction (7). LEA may arise from inadequate dietary intake, excessive exercise energy expenditure, or a combination of both factors and can occur intentionally or unintentionally. Previous research has demonstrated that physiological disturbances may occur when EA falls below approximately 30 kcal·kg^−1^ FFM per day (8, 9). Prolonged or repeated exposure to LEA may contribute to the development of Relative Energy Deficiency in Sport (RED-S), a syndrome characterized by impaired physiological function and performance resulting from insufficient EA (7). The International Olympic Committee (IOC) identifies LEA as the primary etiological factor underlying RED-S and recognizes its effects across multiple physiological systems in athletes of all sexes (7).

The IOC RED-S Physiological Models describe the health and performance consequences associated with chronic LEA (7). From a performance perspective, LEA may impair recovery, reduce training adaptation, and negatively influence physical performance (7). LEA impairs muscle protein synthesis, reduces glycogen availability, and disrupts hormonal signaling necessary for tissue repair and adaptation (5, 7). These disruptions can lead to impaired recovery, decreased muscular strength and power, reduced endurance capacity, and diminished training responsiveness (7). LEA may also impair neuromuscular function and coordination, further compromising performance outcomes (5). Importantly, performance impairments may emerge gradually and may not be immediately apparent, particularly in recreational athletes who lack formal performance monitoring. Recovery represents a critical component of athletic adaptation, and adequate energy availability is essential to support physiological recovery processes. Exercise-induced muscle damage initiates inflammatory and metabolic responses that require sufficient energy and nutrient availability for repair and adaptation (10). Delayed onset muscle soreness (DOMS), characterized by muscle soreness, stiffness, and reduced function, is commonly observed following high-intensity exercise and reflects muscle damage and inflammatory processes (10). LEA may exacerbate recovery impairments by limiting substrate availability for tissue repair and increasing physiological stress (11). Reduced energy availability may also impair immune function, increasing susceptibility to illness and prolonging recovery periods (7).

Subjective measures such as the Rating of Perceived Exertion (RPE), Perceived Recovery Scale (PRS), and Total Quality Recovery (TQR) scale provide practical indicators of training stress, recovery status, and readiness to perform (12–14). Sleep is also an important component of recovery and physiological adaptation, as inadequate sleep has been associated with impaired performance, altered metabolic regulation, and delayed recovery (15, 16). Sleep plays a critical role in muscle protein synthesis, hormonal regulation, and glycogen restoration (16). Sleep deprivation has been shown to impair muscle recovery, reduce endurance performance, and alter metabolic function (16). LEA may negatively influence sleep quality through alteration in endocrine function, including disruptions in cortisol, leptin, and ghrelin regulation, which are critical for appetite and energy balance regulation (17). Sleep disturbances may therefore represent an early physiological consequence of LEA and may contribute to impaired recovery and performance. Additionally, from a health perspective, chronic LEA has also been linked to alteration in bone metabolism, reduced BMD, and increased risk of bone stress injury (7, 17–19). Energy deficiency suppresses reproductive hormone function, particularly estrogen and testosterone, which play essential roles in maintaining bone health (4, 17). Reduced bone mineral density may develop gradually and may remain undetected without clinical assessment. Dual-energy X-ray absorptiometry (DXA) represents the gold standard for assessing bone mineral density and provides an objective measure of skeletal health (20, 21). Together, recovery, sleep, and bone health represent important outcomes within the RED-S framework and may provide insight into the early physiological consequences of inadequate EA.

Despite the growing popularity of HIFT, limited research has examined EA and RED-S risk among recreational HIFT athletes. Most LEA and RED-S investigations have focused on endurance, esthetic, weight-sensitive, or elite athlete populations (5–7, 22–24). As a result, little is known about the prevalence of LEA or its association with recovery, sleep and bone health among recreational HIFT participants. Given the substantial training demands of HIFT and the unique challenges faced by recreational athletes, further investigation is warranted to better understand LEA and its potential physiological consequences within this population.

Therefore, the purpose of this study was to examine energy availability and physiological outcomes consistent with problematic LEA in recreational HIFT athletes within the framework of the IOC RED-S Physiological Models. Specifically, this study aimed to: quantify EA and determine the proportion of athletes meeting LEA criteria; examine associations between EA and recovery-related outcomes, including perceived exertion, and recovery status; and evaluate relationships between EA and health indicators, including sleep quality and bone mineral density. This study extends RED-S research into recreational HIFT populations and provides insight into the potential health and performance consequences associated with LEA in this rapidly growing athletic population.

## Materials and methods

### Study design

This study employed a two-week prospective observational design in which recreational HIFT athletes were monitored over a two-week period to assess energy availability and associated health and performance related measures. The two-week observation period was selected to capture day-to-day variability in dietary intake, exercise energy expenditure, and training/recovery patterns while maintaining participant feasibility and adherence to the intensive monitoring protocol; it was not intended to represent a longitudinal follow-up period or to assess the development of chronic RED-S–related consequences. The PSQI and all demographic information was collected at baseline. The DXA scan was completed on the final days of data collection. Dietary food logs and physical activity logs were completed only on training days. Completion of the PRS and TQR occurred prior to each workout, and RPE was completed following each workout. Exercise energy expenditure was recorded on training days only (5 days per week).

### Participants

Recreational HIFT athletes (*n* = 30; 31.2 ± 8.2 years; females: *n* = 18, weight: 69.9 ± 11.1 kg, height: 164.7 ± 7.1 cm; males: *n* = 12; weight: 89.6 ± 15.1 kg, height: 176.9 ± 6.2 cm) were recruited from local HIFT/CrossFit-style training facilities. Eligibility criteria included: (1) age 18–40 years; (2) participation in ≥5 HIFT sessions per week for at least 6 months prior to enrollment; and (3) engagement in training for general fitness rather than professional or elite competition. Exclusion criteria included: (1) current injury limiting training participation; (2) diagnosed metabolic, endocrine, or bone disorders; and (3) pregnancy or lactation. All participants provided written informed consent prior to participation. All study procedures were conducted in accordance with ethical standards for human subject research at the University of South Carolina’s Institutional Review Board (Pro0060391).

### Baseline instruments

A basic demographic survey that reports age, sex, education level, HIFT participation history, and pertinent health history was used. The timeline of data collection is displayed in Figure 1.

**Figure 1:**
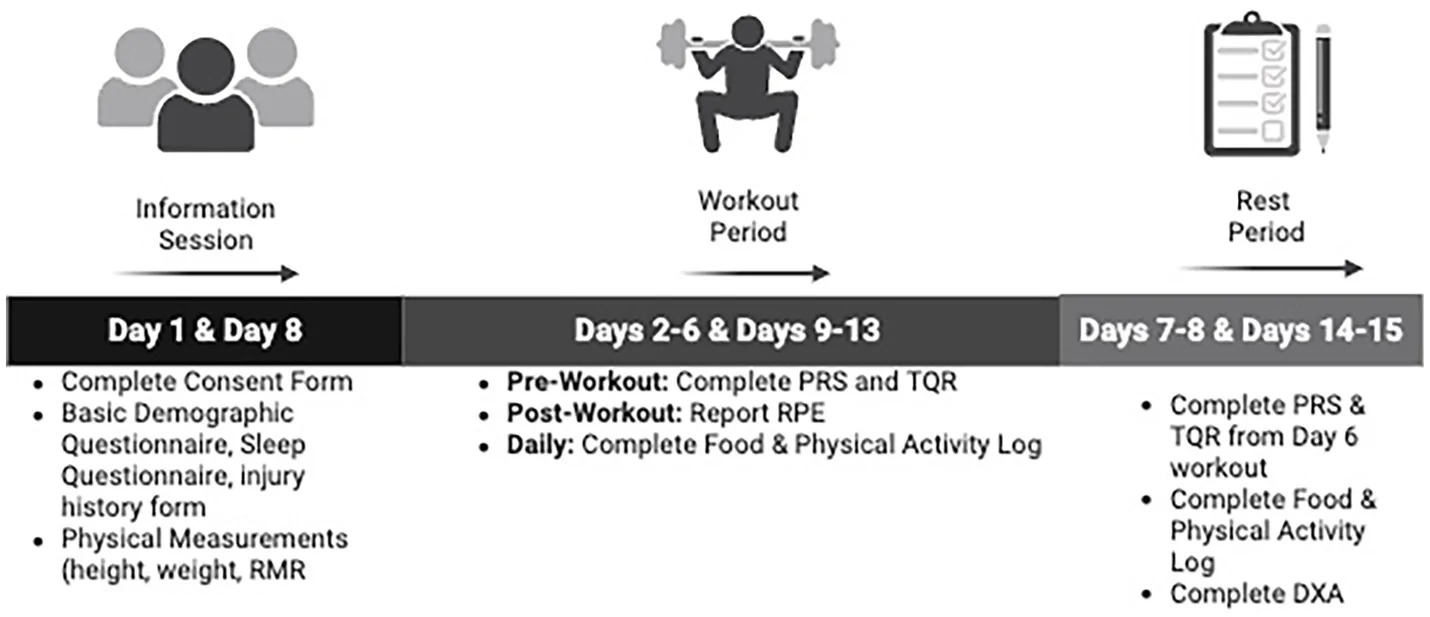
Timeline of data collection. Created in BioRender. Vlattas, G. (2026) https://BioRender.com/iyi9y6p.

### Anthropometric measurement

Anthropometric measurements included height, body mass, and body composition. Height was measured to the nearest 0.1 cm using a portable stadiometer (Shorr Productions, Olney, Maryland, United States). Body mass was measured to the nearest 0.1 kg using a calibrated digital scale (Tanita Corp 331S, Tokyo, Japan) with participants wearing light clothing and no shoes. Body composition and bone health were assessed using dual-energy X-Ray absorptiometry (DXA) (GE Lunar Prodigy densitometer, GE Healthcare, Madison, WI, United States) on the final day of the two-week data collection period. Scans were performed by trained personnel according to standardized manufacturer protocols. Body composition measures included percent body fat and fat-free mass, while bone health was assessed using whole-body BMD. BMD was included as an indicator of skeletal health and clinically relevant outcome associated with chronic LEA (20, 25, 26).

### Resting metabolic rate

Resting metabolic rate (RMR) was measured using indirect calorimetry with the MedGem metabolic analyzer (*Microlife MedGem*; HealtheTech, Golden, CO, United States), a portable device validated for estimating resting energy expenditure (27). Participants were tested in the morning following an overnight fast. Consistent with standardized pre-test procedures for the handheld calorimeter, participants were instructed to refrain from strenuous exercise and caffeine for at least 4 h before (27, 28).

Predicted RMR was calculated using the Cunningham equation, which estimates resting metabolic requirements based on fat-free mass (FFM) (29). Measured RMR was divided by predicted RMR to calculate the RMR ratio, which was used as an indicator of metabolic suppression associated with LEA. Consistent with previous literature, an RMR ratio <0.90 was considered suggestive of physiological adaptations to chronic energy deficiency (7, 11).

### Energy intake and dietary assessment

Dietary intake was assessed using self-reported food logs completed on training day over a two-week period (10 total training days). Participants were instructed to record all foods, beverages, and dietary supplements consumed immediately or as soon as possible after consumption to minimize bias recall. Participants received standardized instructions for completing the dietary records using FoodProdigy (Trustwell, ESHA Research, Beaverton, OR, United States), including recording all foods, beverages, and snacks consumed; documenting brand names and preparation methods when applicable; estimating portion sizes using household measures or package information. Participants were instructed to enter foods as close to the time of consumption as possible to minimize recall error and improve the accuracy of dietary intake estimates. Upon submission, food records were reviewed for completeness and clarification when submitted records contained missing information prior to analysis. Dietary records were entered into FoodProdigy and analyzed using Food Processor Nutrition Analysis Software (version 11.9; Trustwell, ESHA Research, Beaverton, OR, United States) to estimate total daily energy intake (kcals/day) and macronutrient intake (g/day) (30–32). Similar dietary assessment methods have been previously used to evaluate dietary intake and nutritional practices (2).

### Exercise energy expenditure

Exercise energy expenditure (EEE) and Total Daily Energy Expenditure (TDEE) was estimated for each training session and day by the BodyMedia Sensewear armband, a validated multi-sensor device incorporating accelerometry and physiological sensors to estimate energy expenditure in free-living conditions (33–35). In free-living healthy adults, the BodyMedia Sensewear armband demonstrated a mean absolute error of 8.1% ± 6.8% for total energy expenditure compared with doubly labeled water; however, error may be greater during vigorous exercise (33–35). Training sessions were classified according to metabolic equivalent (MET) values and intensity descriptors typical of HIFT programming.

### Energy availability

Energy availability (EA) was calculated for each participant using the following equation: EA = (Energy Intake − Exercise Energy Expenditure)/Fat-Free Mass (kg). This approach is widely used to estimate energy availability in athlete populations (36). Fat-free mass was determined using DXA. EA values were calculated for each training day and averaged across Week 1 and Week 2 (10 total days). LEA was defined as EA < 30 kcal·kg^−1^ fat-free mass·day^−1^, a threshold associated with disruptions in endocrine and metabolic function (4, 8). Participants were categorized as LEA or non-LEA for group comparisons.

### Rating of perceived exertion

Training intensity was assessed using the Borg 6–20 Rating of Perceived Exertion scale, a validated psychophysiological instrument that quantifies an individual’s subjective perception of exercise intensity (12). The Borg RPE scale ranges from 6 (“no exertion at all”) to 20 (“maximal exertion) and has been shown to correlate strongly with physiological indicators of exercise intensity, including heart rate, oxygen consumption, and blood lactate concentration (37). Participants reported their overall session RPE immediately following each training session, with higher scores reflecting greater perceived physical effort. Mean session RPE across the two-week training period was calculated for each participant and used as an indicator of perceived training intensity.

### Perceived recovery scale

Perceived recovery status was assessed using the PRS, a validated subjective measure of an individual’s readiness to perform prior to exercise (13). The PRS consists of an 11-point scale ranging from 0 (very poorly recovered/extremely fatigued) to 10 (very well recovered/highly energetic), with higher score indicating greater perceived recovery and readiness for training. Consistent with previous literature, PRS scores of 8–10 were interpreted as high recovery, 4–7 moderate recovery, and 0–3 poor recovery.

### Total quality recovery

TQR was assessed using the validated 6–20 point TQR scale, a subjective measure of overall recovery status that parallels the Borg Rating of Perceived Exertion Scale (14). The TQR evaluate an athlete’s perceived recovery by integrating multiple physiological and psychological domains, including sleep quality, nutrition, physical restoration, relaxation, emotional well-being, and recovery practices such as stretching or cooldown activities. Higher scores indicate more complete recovery and greater readiness for subsequent training, whereas lower scores reflect inadequate recovery. TQR scores <13 were considered indicative of incomplete recovery (14).

### Sleep assessment

The PSQI is a 19-item scale that is a self-reported measure that allows the participant to recall sleep patterns and habits over a month (38). Responses are used to calculate a global score ranging from 0–21, with higher scores indicating poorer sleep quality. The PSQI has demonstrated good reliability and validity in adult populations (Cronbach’s *α* = 0.83) (38).

### High-intensity functional (HIFT) protocol

Participants completed a standardized HIFT program over a two-week period consisting of five supervised training sessions per week (10 total training sessions). All participants completed the identical training program, with each workout standardized for exercise selection, training volume, intensity, duration, and recovery periods. Sessions incorporated a combination of resistance training, Olympic lifting movements, bodyweight exercises, cardiovascular conditioning, and metabolic circuits representative of contemporary HIFT programing. Workouts targeted both upper- and lower body musculature and included strength, power, muscular endurance, and aerobic conditioning components. All sessions were led by a certified coach to ensure consistency in instruction, exercise technique, and workout implementation across participants.

### Study recruitment and information session procedures

Primary investigators recruited participants from a local HIFT gym through flyers posted in the facility and announcements distributed via affiliated social media platform (e.g., Facebook and gym website). Interested individuals attended an informational session during which the study purpose, procedures, and participant responsibilities were explained. Following the provision of written informed consent, participants completed a demographic questionnaire, injury history questionnaire, and the Pittsburgh Sleep Quality Index (PSQI) using paper-based forms. Participants completed the standardized HIFT training program Monday through Friday for 2 weeks (10 total training sessions) and were instructed to refrain from structured exercise on Saturday and Sundays. Participants arrived approximately 30 min before each training session to complete pre-exercise assessments, including the PRS scale and the TQR scale. Participants also submitted their daily dietary logs, documenting food and beverages consumed on each training day. Following completion of standardized HIFT workout, participants reported their session RPE. On weekends, participants completed the PRS and the TQR assessments only to monitor recovery status while abstaining from structured exercise.

### Statistical analyses

SPSS statistical software (version 31; SPSS Inc., Armonk, NY) was used for all analyses. The present analyses were conducted using an existing dataset comprising 30 participants; therefore, sample size was fixed and not determined by an *a priori* analysis for the current study aims. To characterize the detectable effect size given the available sample, a sensitivity analysis was conducted in G*Power (version 3.1.9.4., Heinrich Heine University, Dusseldorf, Germany) for the primary between-group comparisons. With 24 participants classified as LEA and 6 as non-LEA, a two-tailed independent-samples *t*-test with alpha level of 0.05 and 80% power, the available sample was sufficient to detect a standardized mean difference of approximately Cohen’s *d* = 1.33. As such, the analyses were primarily sensitive to large between-group effects, and smaller effects may not have been detected. Effect-size estimates and 95% confidence intervals are reported to characterize the magnitude and precision of observed effects (39). A significance level was set at *p* < 0.05 for all analysis.

Participant demographic and baseline characteristics, including age, BMD, sleep quality (PSQI global score), energy availability (EA), rating of perceived exertion (RPE), perceived recovery status (PRS), total quality recovery (TQR), and sleep duration, were summarized using descriptive statistics. Continuous variables are presented as means ± standard deviations and 95% confidence intervals (CI). Categorical variables, including sex and LEA classification, are presented as frequencies and percentages. Group differences in demographic and baseline characteristics between sexes and between LEA and non-LEA groups were evaluated using independent samples *t*-tests for continuous variables and chi-square tests of independence for categorical variables. An analysis of covariance (ANCOVA) was conducted to examine BMD differences between LEA and non-LEA participants while adjusting for age as a covariate.

Energy availability (EA) was calculated for each training day as kcal·kg^−1^ fat-free mass·day^−1^. Mean EA was calculated across the two-week observation period. Participants with a two-week mean EA < 30 kcal·kg^−1^ FFM·day^−1^ were classified as LEA, whereas those with a mean >30 kcal·kg^−1^ FFM·day^−1^ were classified as non-LEA. The proportion of participants meeting LEA criteria was determined using frequency analyses. Pearson correlation was used to examine the relationship between continuous mean EA and RMR ratio. Fisher’s exact test was used to examine the association between categorical LEA and RMR-ratio risk classification because of the small, expected cell frequency. For this analysis, we used the average EA across 2 weeks. Sex differences in LEA prevalence were evaluated using Fisher’s exact test because of small, expected cell frequencies. Sex differences in mean EA were evaluated using independent-samples *t*-tests.

Group differences in performance and recovery outcomes, including Rating of Perceived Exertion (RPE), Perceived Recovery Status (PRS), Total Quality Recovery (TQR), and sleep duration, between LEA and non-LEA participants were examined using independent-samples *t*-tests. Mean scores for each scale were calculated across the two-week observation period and used for continuous statistical analyses. Associations between continuous EA and performance and recovery measures were assessed using Spearman rank-order correlations (*ρ*). Sex-based differences in performance and recovery variables were examined using independent-samples *t*-tests. To examine the independent and interactive effects of sex and LEA classification on performance and recovery outcomes, two-way analyses of variance (ANOVA) were conducted. Main effects for sex and LEA, as well as Sex × LEA interaction effects, were evaluated. Partial eta-squared (*η*^2^) was reported as the measure of effect size.

Group differences in health outcomes, including sleep quality (PSQI global score) and BMD *Z*-score, between LEA and non-LEA participants were assessed using independent samples *t*-tests. BMD analyses included 29 participants because an appropriate reference population for calculation of the BMD *Z*-score was unavailable for one participant. Associations between continuous EA and health-related outcomes (BMD *Z*-score and PSQI) were evaluated using Spearman correlations.

Effect sizes were calculated for all primary analyses. Cohen’s *d* was used for independent-samples *t*-tests, Phi coefficients were reported for Chi-square analyses, and partial eta-squared (*η*^2^) was reported for ANOVA and ANCOVA models. For nonparametric associations, Spearman’s *ρ* was interpreted as the effect size. Effect sizes were classified using the following ranges: trivial (*d* < 0.20; |*ρ*| < 0.10), small (*d* = 0.20–0.49; |*ρ*| = 0.10–0.29), moderate (*d* = 0.50–0.79; |*ρ*| = 0.30–0.49), and large (*d* ≥ 0.80; |*ρ*| ≥ 0.50). We examined 95% confidence intervals alongside point estimates and *p*-values to assess precision and clinical relevance independent of statistical significance.

## Results

A total of 37 HIFT athletes inquired about participation in the study. Of these, 30 provided informed consent and enrolled. All 30 enrolled participants completed the study and were included in the final analysis, resulting in no participant withdrawals or losses in follow-up. It should be noted that all 30 participants completed a DXA scan. However, *Z*-scores were available for only 29 participants. A *Z*-scores could not be generated for one participant because of appropriate age- and population-specific reference data set was not available within the DXA software. Therefore, analyses involving *Z*-scores were conducted using the 29 participants for whom *Z*-scores were available. Final participants included 18 females and 12 males with a mean age of the sample was 31.2 ± 8.2 years. Descriptive statistics for all study variables are presented in Table 1.

**Table 1:** Physical measurements for female (n = 18) and male (n = 12) HIFT athletes.

Demographic	All	Females	Males	95% CI	*p*-value
Age (years)	31.2 ± 8.2	30.5 ± 7.9	32.2 ± 8.8	27.7–33.6	0.59
Height (cm)	169.6 ± 9.0	164.7 ± 7.1	176.9 ± 6.2	166.3–173.2	<0.001
Weight (kg)	77.8 ± 15.9	69.9 ± 11.1	89.6 ± 15.1	72.3–84.4	<0.001
Body mass index (kg/m^2^)	26.9 ± 4.1	25.7 ± 3.1	28.6 ± 4.8	25.5–28.5	0.054
Free fat mass (kg)	58.5 ± 13.4	49.2 ± 6.6	72.5 ± 7.3	53.8–64.1	<0.001
Body fat (%)	26.4 ± 7.7	29.2 ± 5.5	22.3 ± 8.8	23.3–29.3	0.01
Bone mineral density (*Z*-score)^a^	1.8 ± 0.88	1.9 ± 0.87	1.8 ± 0.27	1.4–2.1	0.77

a BMD *Z*-score data were available for only 29 participants due to lack of available reference group.

Values are presented in mean ± standard deviation.

### Energy availability

Energy needs for HIFT athletes can be found on Table 2. Mean energy availability (EA) across the two-week observation period was 24.9 ± 9.7 kcal·kg^−1^ fat-free mass (FFM)·day^−1^ (95% CI, 21.2 to 28.6). Twenty-four of 30 participants (80.0%) met criteria for low energy availability (EA < 30 kcal·kg^−1^ FFM·day^−1^). Among females, 15 of 18 (83.3%) were classified as having LEA compared with 9 of 12 males (75.0%). The mean RMR ratio, calculated as measured RMR relative to predicted RMR using the Cunningham equation was 1.10 ± 0.19, exceeding the commonly utilized threshold of <0.90 indicative of metabolic suppression. Pearson correlation analysis demonstrated a positive association between average EA and RMR ratio (*r* = 0.35, CI [−0.01, 0.64], *p* = 0.05, *n* = 30); however, the association did not reach statistical significance. Thus, although higher EA was associated with higher RMR ratios, average EA was not significantly related to RMR ratio in this sample.

**Table 2:** Energy needs for female (*n* = 18) and male (*n* = 12) HIFT athletes.

Demographic	All	Females	Males	95% CI	*p*-value
RMR (kcals)	1975.7 ± 447.5	1761.7 ± 341.6	2296.7 ± 400.3	(−814.2)–(−255.7)	<0.001
Cunningham RMR (kcals)	1787.58 ± 295.69	1582.15 ± 144.18	2095.73 ± 160.45	(−178.9)–93.8	<0.001
RMR ratio	1.10 ± 0.19	1.11 ± 0.19	1.10 ± 0.21	(−0.45)–(−0.13)	0.87
EI week 1 (kcals)	1779.1 ± 620.3	1512.8 ± 389.3	2175.9 ± 701.7	(−1071.1)–(−255.3)	<0.01
EI week 2 (kcals)	1904.6 ± 715.0	1712.6 ± 626.3	2192.7 ± 768.6	(−1003.6)–43.4	0.07
EEE week 1 (kcals)	310.7 ± 63.7	279.3 ± 44.4	357.8 ± 60.3	(−117.6)–(−39.3)	<0.01
EEE week 2 (kcals)	369.1 ± 75.7	331.8 ± 52.7	424.9 ± 71.7	(−139.6)–(−46.7)	<0.01
EA week 1 (kcals/kg FFM)	24.9 ± 10.1	25.0 ± 8.9	24.9 ± 12.1	(−7.7)–8.0	0.97
EA week 2 (kcals/kg FFM)	25.1 ± 9.8	25.2 ± 7.7	25.1 ± 12.8	(−7.5)–7.7	0.99
TDEE (kcals)	2304.9 ± 389.9	2032.9 ± 185.5	2713.1 ± 211.8	(−829.9)–(−530.3)	<0.01

Values are presented in mean ± standard deviation.

Of the 24 participants classified as LEA, 4 (16.7%) also had an RMR ratio indicating energy deficiency, whereas 20 (83.3%) had an RMR ratio ≥ 0.90 and were classified as not at risk. In contrast, none of the 6 participants without LEA had an RMR ratio of <0.90. Thus, all 4 participants demonstrating evidence of metabolic suppression based on RMR ratio were also classified as LEA. Because 50% of the cells had expected counts <5, Fisher’s exact test was used to evaluate the association between LEA and RMR-ratio classifications. There was no statistically significant association between LEA classification and RMR-ratio classification (Fisher’s exact test, two-sided *p* = 0.56). Mean EA did not differ significantly between females (25.1 ± 7.9 kcal·kg^−1^ FFM·day^−1^) and males (24.9 ± 11.9 kcal·kg^−1^ FFM·day^−1^), *t* (28) = 0.03, *p* = 0.98. The mean difference was 0.11 kcal·kg^−1^ FFM·day^−1^ (95% CI: −7.3 to 7.6), indicating negligible between-sex differences (Table 2).

### Performance and recovery outcomes (LEA vs. non-LEA comparisons)

Mean rating of perceived exertion (RPE) was 15.9 ± 1.3 (95% CI: 15.5 to 16.4). Mean perceived recovery status (PRS) was 6.4 ± 0113 (95% CI: 6.0 to 6.8) and mean total quality recovery (TQR) score was 13.7 ± 2.7 (95% CI: 12.6 to 14.7). No statistically significant differences were observed between LEA and non-LEA groups for performance or recovery variables (Table 3). Mean RPE was 15.7 ± 1.3 in LEA participants and 16.5 ± 0.73 in non-LEA participants, *t* (28) = 1.40, *p* = 0.17. The mean difference was 0.81 units (95% CI: −0.4 to 2.0), representing a moderate effect size (*d* = 0.64). PRS was 6.4 ± 1.1 in LEA participants and 6.5 ± 1.- in non-LEA participants, *t* (28) = 0.2, *p* = 0.82, with a trivial effect size (*d* = 0.1). TQR was 13.6 ± 2.9 in LEA participants and 14.1 ± 1.7 in non-LEA participants, *t* (28) = 0.4, *p* = 0.7, with a small effect size (*d* = 0.2). Sleep duration was 6.8 ± 0.7 h/night in LEA participants and 6.8 ± 0.6 h/night in non-LEA participants, *t* (28) = 0.1, *p* = 0.9, with a trivial effect size (*d* = 0.1). Two-way ANOVA models revealed no significant main or interaction effects of sex and LEA classification for RPE (*F*_(1,26)_ = 1.76, *p* = 0.19, *η*^2^ = 0.06), PRS (*F*_(1,26)_ = 0.01, *p* = 0.92, *η*^2^ = <0.01), TQR (*F*_(1,26)_ = 1.41, *p* = 0.25, *η*^2^ = 0.05), or sleep duration (*F*_(1,26)_ = 0.01, *p* = 0.94, *η*^2^ < 0.001). There were no statistically significant sex differences in RPE, PRS, TQR, or sleep duration. Between-sex effect sizes were trivial to small for RPE, TQR, sleep duration, PSQI, and BMD, whereas the effect size for PRS was moderate (*d* = −0.57) (Table 4).

**Table 3:** RED-S performance model outcomes by LEA status.

Outcome	LEA	Non-LEA	*t* (d*f*)	*p*	Cohen’s *d*	Effect size	95% CI
RPE	15.7 ± 1.3	16.5 ± 0.7	1.4 (28)	0.17	0.64	Moderate	−0.4 to 2.0
PRS	6.4 ± 1.1	6.5 ± 1.0	0.2 (28)	0.682	0.10	Trivial	−0.91 to 1.14
TQR	13.6 ± 2.9	14.1 ± 1.7	0.4 (28)	0.67	0.19	Trivial	−2.0 to 3.1
Sleep duration	6.8 ± 0.7	6.8 ± 0.6	0.1 (28)	0.94	0.10	Trivial	−0.6 to 0.6

Values are mean ± SD with 95% confidence intervals (CI). RED-S performance model variables include RPE, PRS, and TQR.

**Table 4:** Sex-stratified comparisons of RED-S performance and health variables.

Variable	All (*n* = 30)	Female (*n* = 18)	Male (*n* = 12)	95% CI	*p*
RPE	15.9 ± 1.3	15.8 ± 1.3	15.9 ± 1.3	15.5–16.4	0.95
PRS	6.4 ± 1.1	6.2 ± 1.1	6.8 ± 0.9	6.0–6.8	0.14
TQR	13.7 ± 2.7	13.6 ± 2.7	13.7 ± 2.7	12.6–14.7	0.97
Sleep duration (h)	6.8 ± 0.64	6.8 ± 0.74	6.8 ± 0.50	6.5–7.0	0.94
PSQI global score	6.3 ± 2.7	6.5 ± 2.3	6.1 ± 3.3	5.3–7.4	0.69
BMD (*Z*-score)^a^	1.8 ± 0.88	1.9 ± 0.87	1.8 ± 0.27	1.5–2.2	0.77

Values are mean ± SD. Independent samples *t*-tests conducted between sexes. RPE, rating of perceived exertion; PRS, perceived recovery status; TQR, total quality recovery; BMD, bone mineral density.

a BMD Z-Score data were available for only 29 participants due to lack of available reference group.

### Associations with energy availability

Spearman correlation analyses revealed no significant associations between EA and RPE (*ρ* = 0.16, *p* = 0.39), PRS (*ρ* = −0.07, *p* = 0.39), TQR (*ρ* = 0.19, *p* = 0.29), or sleep duration (*ρ* = 0.03, *p* = 0.86) (Table 5). In an exploratory analysis, sleep duration was moderately positively associated with TQR (*ρ* = 0.46, *p* = 0.01). No adjustment for multiple comparisons was applied; therefore, this finding should be interpreted cautiously.

**Table 5:** Associations between energy availability and RED-S performance variables.

Variables	Spearman *ρ*	95% CI	*p*	Magnitude
EA and RPE	0.16	(−0.2)–0.5	0.39	Small
EA and PRS	−0.07	(−0.5)–0.4	0.72	Trivial
EA and TQR	0.20	(−0.2)–0.5	0.29	Small
EA and sleep duration	0.01	(−0.3)–0.4	0.86	Trivial
EA and BMD (*Z*-score)	−0.10	(−0.5)–0.3	0.59	Trivial
EA and PSQI	−0.01	(−0.4)–0.4	0.85	Trivial
Sleep duration and TQR	0.5	0.1–0.7	0.01	Moderate

### Health outcomes (LEA vs. non-LEA comparisons)

Mean sleep duration was 6.8 ± 0.6 h per night (95% CI: 6.5 to 7.0), and mean Pittsburgh Sleep Quality Index (PSQI) global score was 6.2 ± 2.7 (95% CI: 5.2 to 7.3). Mean BMD *Z*-score, available for 29 participants, was 1.82 ± 0.88 (95% CI: 1.5 to 2.1). There were no statistically significant differences between LEA and non-LEA groups for BMD *Z*-score or PSQI global score (Table 6). Mean BMD *Z*-score was 1.89 ± 0.87 in LEA participants and 1.53 ± 0.94 in non-LEA participants, *t* (27) = −0.89, *p* = 0.383 (*d* = 0.40, small–moderate). Mean PSQI global scores were 6.42 ± 2.87 (LEA) and 6.00 ± 2.10 (non-LEA), *t* (28) = −0.33, *p* = 0.74 (*d* = 0.17). EA was not significantly associated with BMD *Z*-score (*ρ* = −0.10, *p* = 0.59) or PSQI global score (*ρ* = −0.04, *p* = 0.85), as shown in Table 5. Inclusion of sex i the model did not alter findings, and no significant sex × LEA interaction was observed (*p* = 0.884). Figure 2 demonstrates a graphical summary of RED-S conceptual alignment.

**Table 6:** RED-S health model outcomes by LEA status.

Outcome	LEA	Non-LEA	Statistic	*p*	Effect size	Interpretation	95% CI
Bone mineral density (*Z*-score)	1.9 ± 0.9	1.5 ± 0.9	*t* (27) = −0.9	0.38	*d* = 0.40	Moderate	−1.2 to 0.5
PSQI global score	6.4 ± 2.9	6.0 ± 2.1	*t* (28) = −0.3	0.74	*d* = 0.20	Small	−3.0 to 2.2

Values are mean ± SD with 95% confidence intervals (CI). BMD, bone mineral density; LEA, low energy availability; PSQI, Pittsburgh Sleep Quality Index. BMD *Z*-score data were available for only 29 participants due to lack of available reference group.

**Figure 2:**
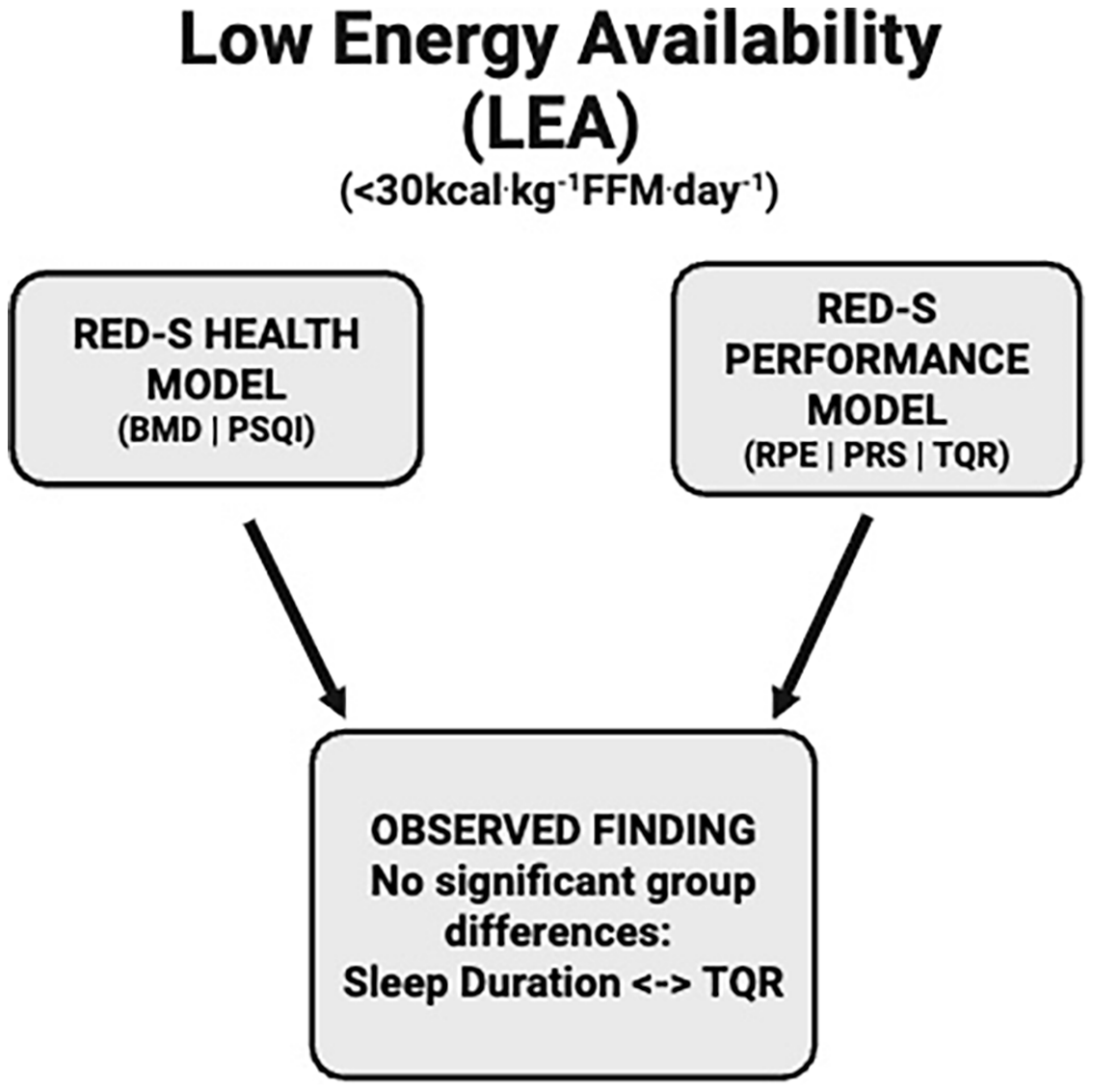
Graphical summary of RED-S conceptual alligment. Created in BioRender. Vlattas, G. (2026) https://BioRender.com/qk617dd.

### Summary of results

Eighty percent of recreational HIFT athletes met criteria for low energy availability. Despite this high prevalence, LEA status was not associated with statistically significant differences in performance, recovery, sleep quality, or bone mineral density. Effect sizes were generally small, and confidence intervals were wide. An exploratory analysis identified a moderate positive association between sleep duration and perceived recovery, suggesting sleep may play an independent role in recovery irrespective of energy availability status, but this finding should be interpreted cautiously.

## Discussion

This study examined the relationship between EA and health and performance outcomes aligned with the RED-S Health and Performance Models in recreational HIFT athletes. Although a substantial proportion of participants demonstrated EA values consistent with LEA risk, LEA was not associated with measured health, recovery, or performance-related outcomes. Importantly, the additional examination of RMR ratio provided a complementary physiological perspective: evidence of potential metabolic suppression was uncommon, and EA was not significantly associated with RMR ratio. Collectively, these findings suggest that low calculated EA did not consistently coincide with physiological or perceptual consequences in this sample. This distinction is important because physiological responses to LEA may vary according to the magnitude and duration of energy deficit, prior energy status, and individual susceptibility and different RED-S outcomes may emerge at different rates (7). Thus evidence that some metabolic or endocrine alterations can occur rapidly under control energy-deficient conditions does not necessarily imply that perceptual recovery, sleep, bone health, or other downstream outcomes will change concurrently or within the same timeframe. In the present study, perceived recovery was more closely associated with sleep duration than with EA, further suggesting that short-term recovery perceptions may be influenced by factors other than energy status alone. Rather than indicating that 2 weeks is insufficient for LEA to produce any physiological responses, the present findings demonstrate that LEA classification during this 2-week observational period was not consistently accompanied by the specific physiological, health, and perceptual alterations assessed. These findings reinforce the RED-S framework as a continuum in which the manifestation and timing of health and performance consequences may vary across individual outcomes.

### Interpreting the findings within the RED-S health and performance models

The IOC RED-S model identifies LEA as the central exposure that drives downstream impairments across multiple physiological systems and performance domains (7). The 2023 IOC consensus update further refined the conceptualization of RED-S with distinct and complementary Health and Performance Models, emphasizing that impairments may occur across a spectrum of severity and may not be immediately apparent clinically (7). The Health Model prioritizes longer-term or systemic consequences (e.g., endocrine function, bone health, immunity, sleep disturbance), whereas the Performance Model highlights training adaptation, recovery capacity, psychological function, and injury/illness risk as key performance-related outcomes (7). Within this framework, LEA represents the etiological driver of downstream dysfunction.

A core RED-S principle is that physiological systems respond to low energy availability through a combination of short-term adaptive mechanisms and longer-term maladaptive consequences with sustained or repetitive exposure (7). Controlled studies demonstrate that metabolic and endocrine shifts can occur within days of energy restriction, including reductions in leptin, triiodothyronine (T3), and alterations in reproductive hormone pulsatility (1, 8). These early endocrine changes are often interpreted as energy-conserving adaptations. Over longer periods, persistent LEA can contribute to suppressed bone formation, altered neuromuscular function, impaired recovery, and increased injury risk (7, 17–19). A two-week observational window, particularly in a free-living recreational cohort, may capture a period in which compensatory mechanisms maintain functional outcomes despite low calculated EA. This interpretation is supported by the findings that EA was not meaningfully associated with the measured health or performance outcomes (15, 40), whereas longer sleep duration was moderately associated with higher TQR scores, suggesting that sleep may rt. a more immediate influence on perceived recovery than short-term fluctuations in EA. However, this association was exploratory and should be interpreted cautiously considering these findings cannot establish a causal effect of sleep on recovery.

### LEA prevalence in recreational HIFT athletes: implications and context

A notable finding was that 80% of participants demonstrated calculated EA values <30 kcal·kg^−1^ FFM·day^−1^, threshold used to classify LEA in this study. This cutoff was selected based on foundational EA research demonstrating physiological alterations below approximately this level and its subsequent use in the operational thresholds in consensus statements (5–7). However, <30 kcal·kg^−1^ FFM·day^−1^ should not be interpreted as a universal diagnostic threshold, as physiological responses to LEA vary across individuals and the cutoff has not been specifically validated in recreational HIFT athletes. This distinction is particularly relevant given the intermittent, mixed-modality nature of HIFT and variability in training energy expenditure. Nevertheless, inadequate EA remains relevant in this population because HIFT combines substantial training demands with potential leanness-oriented and performance-esthetic goals that may contribute to intentional or inadvertent under-fueling (7, 11, 22, 24). Previous studies in CrossFit/HIFT populations have similarly identified potential mismatches between energy intake and training demands, and a tendency toward under-consumption of total energy or key macronutrients in some subgroups (2, 3, 41). Thus, the high proportion of participants below 30 kcal·kg^−1^ FFM·day^−1^ identifies potentially inadequate EA rather than confirming physiologically consequential LEA or RED-S. Physiological responses may vary according to sex, training demands, magnitude and duration of energy deficit and individual susceptibility. Thus, supporting the need to interpret calculated EA alongside physiological and clinical indicators.

Despite the high frequency of LEA based on calculated EA, the mean RMR ration remained above the threshold commonly used to indicate metabolic suppression. Average EA was positively associated with RMR ration calculated using the Cunningham question (*r* = 0.354), suggesting that lower EA generally corresponded with lower RMR ratios; however, this relationship did not reach statistical significance (*p* = 0.055). These findings suggest that calculated EA and RMR ratio may capture different aspects of energy status and should not be expected to correspond uniformly at the individual level. The absence of a statistically significant association may also reflect the duration and magnitude of energy deficiency experienced by participants. RMR suppression represents a physiological adaptation that may develop in response to more prolonged or substantial energy deficiency, whereas the present study assessed EA over a two-week period. Consequently, low EA observed during the monitoring period may not necessarily indicate that participants had experienced energy deficiency of sufficient duration or severity to produce measurable suppression of RMR. Additionally, measurement error inherent in estimates of energy intake and exercise energy expenditure may attenuate the relationship between calculated EA and physiological indicators of energy status. These findings reinforce the importance of interpreting calculated EA alongside physiological and clinical indicators rather than relying on a single EA threshold to characterized energy deficiency.

Importantly, EA estimation is sensitive to both EI and EEE measurement error (36). While this study used structured dietary logging and objective EEE assessment via an armband (an advantage relative to fully self-reported EEE), self-reported food logging can still underestimate true intake, especially in physically active individuals (30–32). Underreporting could inflate LEA prevalence by lowering estimated EI and thus EA (30, 31). The relatively low reported energy intake observed in this sample further warrants consideration of potential dietary underreporting. This is particularly relevant among male participants, whose reported mean energy intake was approximately 2,175–2,193 kcal·day^−1^, despite a measured mean RMR of approximately 2,296 kcal·day^−1^. Consequently, the high prevalence of calculated LEA observed may reflect a combination of true inadequate energy availability and measurement error associated with self-reported dietary intake. In addition, armband-based EEE estimates, while more objective than self-reporting measures, are not equivalent to gold-standard calorimetry and may introduce bias depending on activity type, placement, and algorithm assumptions (33–35). As SenseWear accuracy may decline at higher exercise intensities, the corresponding EEE estimates should be interpreted cautiously in HIFT sessions containing vigorous, intermittent, and mixed-modality activity (34, 35). Collectively, these methodological considerations do not negate the observation that a substantial portion of this sample demonstrated calculated EA below the established threshold; rather, they warrant caution in interpreting the exact proportion as representative of the broader recreational HIFT population. The relatively low occurrence of suppressed RMR ratios and lack of a significant relationship between EA and RMR ratio further underscore the value of evaluating EA alongside complementary physiological and clinical indicators, including RMR suppression, endocrine function, menstrual or reproductive function, bone health, and injury history (7, 36). Future studies with larger samples and longitudinal designs are needed to determine whether sustained LEA in recreational HIFT athletes is associated with subsequent metabolic adaptations or other RED-S related health and performance consequences.

### Performance outcomes: why might LEA not translate to measurable differences in two weeks?

Under the RED-S Performance Model, LEA is expected to compromise training response and recovery, which could manifest as increased perceived exertion, reduced perceived readiness, poorer recovery behaviors, or decreased performance output (7). In this cohort, none of the key perceptual performance indicators differed significantly between LEA and non-LEA groups, and continuous EA was not correlated with RPE, PRS, or TQR. The absence of clear differences in perceived exertion and recovery should be considered within the context of variable performance responses to LEA reported in the literature. Although prolonged or more severe LEA has been associated with impaired training response and performance, these effects may vary according to the magnitude and duration of energy deficiency, athlete population, and outcome assessed (7, 11). This variability may be particularly relevant to the present study because RPE, PRS, and TQR reflect subjective perceptions of exertion, readiness, and recovery rather than direct measures of exercise performance (12–14). Accordingly, the absence of associations between calculated EA and these perceptual outcomes does not necessarily indicate an absence of physiological or performance-related effects of LEA (7).

Several explanations are plausible. First, the HIFT modality may be less sensitive to short-term energy deficits in terms of subjective exertion and perceived recovery compared with endurance performance outcomes. HIFT includes intermittent high-intensity efforts and strength/power components that may be buffered in the short term by glycogen availability, neuromuscular familiarity, motivation, caffeine use, or pacing strategies (1, 41, 42). LEA has been studied extensively in endurance athletes, in whom high training expenditures and prolonged exercise may increase the likelihood that inadequate energy and carbohydrate availability affect training and recovery (7, 11). Recreational HIFT athletes may also modulate their training intensity across sessions (intentionally or unconsciously), thereby maintaining perceived exertion within a stable range even if training load fluctuates. The Borg RPE scale also reflects perceived intensity, not necessarily objective output, and may remain stable despite subtle declines in performance if athletes adjust pace accordingly (12). Markedly, the direction of the RPE group difference was not consistent with the expected pattern under the RED-S framework, as participants classified as LEA reported lower RPE than non-LEA participants (15.7 vs. 16.5). However, this difference was not statistically important, and the small non-LEA group (*n* = 6) limits the precision of this estimate. Therefore, the observed direction should not be interpreted as evidence that LEA is associated with lower perceived exertion.

Notably, the mean PRS score for the overall sample (6.4 ± 1.1) fell within the range categorized as moderate perceived recovery (4–7). At the participant level, 28 participants (93.3%) had mean PRS values within the moderate recovery range, while two participants (6.7%) had mean values within the high recovery range; no participants had a mean PRS within the poor recovery range. PRS did not differ between participants with and without LEA and was not associated with continuous EA, suggesting that perceived recovery during the two-week observation period was not clearly differentiated by energy availability in this population. Participants completed five HIFT sessions per week, and the cumulative demands of frequent high-intensity training may contribute to variability in perceived recovery. However, because training load and other potential determinants of recovery were not independently evaluated, the factors contributing to the low PRS scores cannot be determined from the present study. These findings highlight the importance of considering recovery alongside EA when evaluating recreational HIFT athletes.

Second, RED-S performance impairments may emerge more clearly with chronic exposure or higher-severity LEA. Although endocrine responses to reduced EA can occur within days following reduced EA, the time course over which these changes translate into detectable performance impairment remains uncertain and likely varies according to the severity and duration of LEA and the outcome measured (7, 8). The present study’s short observational duration may not have allowed sufficient time for LEA-related decrements to accumulate into detectable differences in perceptual scales. Third, the unequal group sizes (LEA *n* = 24, non-LEA *n* = 6) reduce power for between-group comparisons. Collectively, the direction of the point estimates was not uniformly consistent with LEA-related impairment, reinforcing the uncertainty of between-group comparisons in this small sample. Wide confidence intervals across outcomes indicate imprecision and elevate the likelihood of Type II error. Even when effect sizes appear small-to-moderate, the study may be underpowered to declare statistical significance. This is particularly relevant in pragmatic recreational studies, where true effects may be modest and distributed across individuals rather than manifesting as large mean differences.

Finally, the selected outcomes may not be the most sensitive early markers of LEA in HIFT. The RED-S Performance Model includes outcomes like decreased training response, impaired coordination, increased injury risk, and mood/psychological changes (17). While RPE, PRS, and TQR are widely used and practical, they are subjective and may have limited sensitivity to subclinical physiological changes (13, 14). Incorporating objective performance metrics (e.g., standardized WOD time, power output, bar velocity, jump height), training load quantification, or biomarkers of recovery and inflammation could help detect early physiological perturbations (43, 44).

### Sleep and recovery: exploratory associations

In an exploratory analysis, sleep duration was moderately positively associated with TQR (*ρ* = 0.460). This association is consistent with a robust body of literature linking sleep duration/quality to recovery, perceived readiness, and performance in athletes (15, 40, 44–46). Sleep contributes to physiological recovery, cognitive function, mood regulation, and readiness for exercise, whereas sleep loss may impair reaction time, endurance, perceived effort, and other performance-related outcomes (15, 40, 47). In athletic cohorts, sleep extension interventions have improved subjective recovery and performance-relevant outcomes, supporting a potentially causal role for sleep (43, 44). However, the present study cannot establish a causal effect of sleep on recovery. In contrast, LEA status was not associated with sleep duration or PSQI score, and EA was not correlated with PSQI. These null findings may reflect the short timeframe and the multifactorial nature of sleep in athletes, including the influence of training schedules, stress, competition demands, and habitual sleep behaviors (16, 43, 45, 46). Additionally, the PSQI assesses global sleep quality over the preceding month (38). Accordingly, the PSQI score may primarily represent habitual sleep before and during the study rather than sleep changes occurring specifically within the two-week observation period (38).

Within RED-S models, sleep disturbance is listed as a potential health consequence, but evidence linking LEA directly to sleep outcomes remains emerging and may differ by severity, duration, and population (7). The present findings suggest a potential relationship between sleep duration and perceived recovery in recreational HIFT athletes. However, this finding should be considered exploratory, and support continued attention to sleep and recovery behaviors alongside fueling.

### Bone health: time course and interpretation in a short observational study

The RED-S Health Model emphasizes bone health as a key long-term outcome affected by chronic LEA (7). In this study, BMD *Z*-score did not differ significantly by LEA group and was not associated with EA. The lack of significant association between EA and BMD Z-score should be interpreted in the context of the complex relationship between energy availability and skeletal health. Prolonged LEA and RED-S related physiological disruption have been associated with adverse skeletal outcomes, including altered bone metabolism and increased bone injury risk (7, 17–19). However, interpretation of calculated EA relative to skeletal outcomes may be influenced by differences in EA assessment methods, duration and severity of energy deficiency, and the cumulative nature of BMD as an indicator of skeletal status (7, 17, 18, 36). Additionally, the resistance and impact loading characteristic of HIFT may provide an osteogenic stimulus that influences BMD independently of EA status (20, 25). In the present population, the higher BMD *Z*-score among participants classified as LEA should therefore not be interpreted as a benefit of LEA, particularly given the small non-LEA group and absence of statistically significant difference. This pattern is unsurprising given the biology of bone remodeling. Bone mineral density changes slowly, typically requiring months to years to manifest measurable differences, whereas metabolic or endocrine changes may occur within days or weeks (17–19). Controlled LEA studies demonstrate that bone formation markers may decline within short periods, but BMD itself is a cumulative endpoint reflecting longer-term remodeling and mechanical loading (17–19). Therefore, null between-group BMD differences should not be interpreted as evidence that LEA is benign for bone health in HIFT athletes. Rather, BMD in a short-term observational study is more appropriately interpreted as an indicator of baseline skeletal status and potential cumulative exposure rather than an outcome expected to change over 2 weeks (7).

Importantly, HIFT includes resistance loading and impact elements that may exert osteogenic stimuli, potentially supporting BMD maintenance despite LEA risk (20, 25). Resistance training has been associated with positive effects on bone density and structure, though adequate nutrition and hormonal milieu remain important for optimal adaptation (20, 25). A plausible hypothesis is that mechanical loading in HIFT may partially counteract skeletal risks in the early phases, with potential LEA-related detriments emerging over longer time horizons or in subgroups with severe restriction, menstrual dysfunction, or low vitamin D/calcium intake (5, 7).

Including age as a covariate did not change findings. Age influences BMD and could confound associations in mixed-age samples (21). The age-adjusted models show non-significant LEA effects, suggesting either a true null relationship in this short timeframe or insufficient power/contrast to detect an effect. Future studies could strengthen inference by including sensitive markers of bone turnover (e.g., P1NP, CTX), menstrual function assessments, vitamin D status, and history of stress injuries, which may detect early bone-related effects more effectively than BMD alone (5, 17–19).

### Sex differences and the role of distributional imbalance

No significant sex differences were observed in EA, performance/recovery measures, sleep, or BMD, and sex-by-LEA interaction effects were non-significant. This finding aligns with the observation that LEA prevalence was high in both sexes, limiting contrast. Historically, research emphasized the Female Athlete Triad, but RED-S and the Male Athlete Triad broadened the focus to include males and diverse sports (6, 7). The RED-S framework increasingly recognizes that LEA affects males as well as females, including impacts on testosterone, other metabolic hormones, and performance (7).

That said, sex-specific pathways may still exist, particularly regarding reproductive endocrine function and bone remodeling (5, 7, 8). In women, energy deficiency can disrupt gonadotropin-releasing hormone pulsatility and reduce estrogen, which has downstream effects on bone and vascular health; a classic threshold relationship between EA and luteinizing hormone pulsatility was demonstrated in controlled studies (8). In men, LEA has been associated with suppressed testosterone and altered metabolic hormones, though the dose–response and clinical thresholds may differ (26, 48). Without endocrine biomarkers or menstrual function data, sex-specific RED-S mechanisms cannot be directly evaluated here. The lack of sex differences in the current outcomes thus should not be interpreted as evidence that sex-specific physiological responses are absent; rather, they may not be detectable in the current design and outcome set.

### Confidence intervals, precision, and underpowering

A consistent feature of these results is that many confidence intervals were wide and crossed zero. This indicates imprecision and supports a cautious interpretation of null findings. In a sample of 30 with an 80/20 group split, the smallest detectable effects for between-group comparisons are likely moderate-to-large. Therefore, the study could miss small-to-moderate true effects, especially if individual variability is high. This is a common challenge in pragmatic field studies of recreational athletes. The implications are that while the data does not support strong short-term LEA effects on the measured outcomes, the results do not rule out clinically meaningful effects that may emerge with larger samples, longer observation, or more sensitive endpoints. Interpreting confidence intervals as plausible ranges of effects aligns with contemporary reporting standards and reduces overreliance on *p*-values (49, 50). In practice, the combination of high LEA prevalence and uncertain short-term effects underscores the need for prospective longitudinal studies rather than concluding that LEA is inconsequential in recreational HIFT athletes.

### Methodological considerations: EA assessment and misclassification risk

EA estimation in free-living contexts remains challenging (36). The calculation depends on accurate EI and EEE measurement and an appropriate FFM estimate. While DXA-based FFM improves precision relative to anthropometric estimates, EI measurement via food logs is vulnerable to both random and systematic error, including underreporting, reactivity, and portion size mis-estimation (30–32). In addition, training-day-only logging can miss compensatory intake patterns on rest days, which may contribute meaningfully to weekly energy balance and recovery (36). Similarly, even though armband-based EEE provides objective estimation, wearable algorithms can vary in accuracy by activity type, intensity, and movement pattern (33–35). In mixed-modality training with rapid transitions, external load, and non-steady-state movement, estimation error may increase (34, 35). These issues can result in misclassification (i.e., labeling an athlete as LEA when true EA is adequate, or vice versa) (36), which generally biases results toward the null and could partially explain the lack of associations. Future studies could reduce misclassification by extending monitoring duration, incorporating doubly labeled water for total energy expenditure where feasible, applying standardized dietary assessment protocols with interviewer-assisted recalls, and integrating physiological markers (e.g., RMR suppression, endocrine measures) to validate LEA risk categorization (7, 36).

### Practical implications for recreational HIFT athletes and clinicians

The high proportion of participants with calculated EA below 30 kcal·kg^−1^ FFM·day^−1^ warrants attention but should not be interested as evidence that this threshold along identifies clinically meaningful RED-S risk in recreational HIFT athletes. In the present study, EA classification as not associated with the measured health, recovery, or performance-related outcomes, and evidence of metabolic suppression based on RMR ratio was uncommon. Accordingly, these findings do not support using short-term calculated EA or the <30 kcal·kg^−1^ FFM·day^−1^ threshold as a stand-alone screening tool in this population. Rather, calculated EA may provide one component of a broader assessment that considers physiological and clinical indicators, including menstrual or reproductive health, injury and bone health history, psychological factors, eating behaviors, and changes in performance or recovery (7, 26, 48). For recreational HIFT athletes, education regarding adequate fueling remains appropriate given the substantial and variable energy demands of HIFT and previously reported concerns regarding dietary adequacy in this population (2, 3, 51). However, intervention decision should not be based solely on an EA classification in the absence of corroborating clinical or physiological evidence. Similarly, the observed association between sleep duration and perceived recovery supports attention to sleep and recovery behaviors. As part of a comprehensive athlete health assessment (15, 40, 45, 47). Future longitudinal research is needed to determine whether repeated or sustained LEA is associated with subsequent changes in physiological, health, or performance outcomes and whether <30 kcal·kg^−1^ FFM·day^−1^ threshold provides meaningful discriminatory value in recreational HIFT athletes.

### Strengths, limitations, and future directions

A key strength of this study is the application of RED-S conceptual models to a recreational HIFT population, which is underrepresented in the LEA literature related to endurance and elite sport. Another strength is the use of DXA-derived body composition and objective EEE estimation, which improves EA estimation fidelity compared with purely self-reported methods. Nonetheless, several limitations constrain inference. The sample size was modest, and group distribution was imbalanced, thus limiting power and precision. Additionally, the unequal LEA and non-LEA group sizes limited statistical power for between-group comparisons. Sensitivity analysis indicated that the achieved sample provided 80% power to detect only very large effects (*d* ≥ 1.33); therefore, smaller but potentially meaningful differences may not have been detected. Selection bias is also possible because individuals who volunteered for the study examining EA and health may differ in the nutrition, training, or health behaviors from the broader recreational HIFT population, limiting generalizability. Additionally, participants were aware that their dietary intake and physical activity were being monitored, which may prompt temporary changes in eating or exercise behaviors during the observational period. Such reactivity could result in measured EA that does not fully reflect participants’ habitual EA.

Future research should prioritize longitudinal designs that track EA, endocrine markers (i.e., leptin, T3, cortisol, sex hormones), bone turnover markers, objective performance indicators, and injury incidence over months rather than weeks (7, 17–19). Additionally, the two-week observational design does not establish the duration or chronicity of LEA before or during the observation period. Therefore, these present findings cannot determine the temporal relationship between LEA exposure and the development of RED-S related health or performance consequences. Repeated assessments may also help determine whether short-term EA measurements reflect habitual energy status and reduce the influence of participant reactivity to monitoring. Additional inclusion of validated RED-S screening tools and psychosocial measures (i.e., eating behaviors, body image pressures, stress) would further contextualize LEA risk in recreational HIFT settings (7). Furthermore, intervention studies testing fueling education or sleep optimization strategies in HIFT athletes could determine whether improving these factors enhances recovery, training adaptation, and health markers (15, 40, 45–47).

## Conclusion

In a cohort of recreational HIFT athletes, LEA risk was highly prevalent, with mean EA below the commonly used threshold and 80% of participants classified as at risk. Over the short observational window, LEA was not associated with significant differences in perceptual performance outcomes, sleep, or BMD, and continuous EA showed weak associations with these measures. An exploratory positive association was observed between sleep duration and perceived recovery, potentially suggesting sleep as a critical recovery determinant independent of EA. Within the RED-S framework, these findings suggest that short-term LEA may be common and clinically silent in recreational HIFT athletes, with potential downstream consequences more likely to emerge over longer exposure periods or in the presence of additional risk factors. Continued research using longer follow-up and physiologic markers is required to determine whether sustained LEA in HIFT populations produces the health and performance impairments predicted by the RED-S Health and Performance Models (7).

## Data Availability

The raw data supporting the conclusions of this article will be made available by the authors, without undue reservation.

## Glossary

ACSM: American College of Sports Medicine
ANCOVA: analysis of covariance
BMD: bone mineral density
BMI: body mass index
CI: confidence interval
DXA: dual-energy X-ray absorptiometry
EA: energy availability
EEE: exercise energy expenditure
EI: energy intake
FFM: fat-free mass
HIFT: high-intensity functional training
IOC: International Olympic Committee
LEA: low energy availability
PRS: perceived recovery status
PSQI: Pittsburgh Sleep Quality Index
RED-S: relative energy deficiency in sport
RMR: resting metabolic rate
RPE: rating of perceived exertion
SD: standard deviation
TQR: total quality recovery
*d*: Cohen’s *d* effect size
*F*: *F*-statistic
*M*: mean
*n*: sample size
*p*: probability value
*r*_s_: Spearman rank correlation coefficient
*t*: student’s *t*-statistic
*χ* ^2^: Chi-square statistic
*η* ^2^: Eta squared effect size
cm: centimeters
g: grams
h: Hours
kcal: kilocalories
kg: kilograms
kg·m^−2^: kilograms per square meter
kcal·kg FFM^−1^·day^−1^: kilocalories per kilogram of fat-free mass per day
min: minutes
%: percent
